# The role of government in the achievement of medicines’ security: A preliminary exploration of stakeholders’ views and experience

**DOI:** 10.1371/journal.pone.0299978

**Published:** 2024-06-07

**Authors:** Obi Peter Adigwe, Godspower Onavbavba

**Affiliations:** National Institute for Pharmaceutical Research and Development, Federal Capital Territory, Abuja, Nigeria; Caleb University, NIGERIA

## Abstract

Medicines are essential commodities that form the cornerstone in majority of processes and interventions aimed at assuring optimal healthcare and wellbeing for any population. Apart from being saddled with the responsibility of providing medications for this purpose, the pharmaceutical industry has the potential to catalyse socioeconomic development such as job creation and revenue generation. This study aimed at assessing government’s role in driving development in Nigeria’s pharmaceutical sector. Questionnaires were administered to healthcare practitioners that participated in an event targeted at developing Nigerian pharmaceutical sector. Data collected were analysed using Statistical Package for Social Sciences. A total of 76 respondents participated in the study. Two-thirds of the study participants (69.7%) were males, slightly above a third of the study participants (38.2%) were aged 51 and above, and close to a quarter of the participants (21.1%) were doctorate degree holders. About half of the study participants (51.4%) indicated that Nigerian pharmaceutical sector was not adequately regulated, whilst almost all (97.4%) indicated that engaging the legislature was critical for the development of the sector. A strong majority of the study participants (87.5%) indicated that existing drug laws should be reviewed so as to protect the pharmaceutical sector. Also, majority of the participants (56.3%) were not satisfied with government’s efforts in developing the pharmaceutical industry. Although this study explored a small cohort, its findings have revealed novel insights regarding factors limiting the requisite prioritisation of the Nigerian pharmaceutical sector. The emergent evidence can begin to underpin proactive policy and practice reforms aimed at achieving medicines’ security in Nigeria. Further studies can build on these preliminary findings to enable robust and comprehensive sectoral interventions that improve access to healthcare, whilst also catalysing socioeconomic development.

## Introduction

The pharmaceutical industry is an important sector that plays a prominent role in healthcare provision for the citizenry [1]. Despite its acknowledged critical role [2], the Nigerian pharmaceutical manufacturing sector continues to face significant challenges that limit its capacity to optimally contribute to national healthcare delivery [3]. This is further exacerbated by unfair competition of imported pharmaceutical products, especially for those which local manufacturers have the capacity to produce [4]. Inadequate access to raw materials, lack of cutting-edge technology, and poor power supply are other challenges faced by local pharmaceutical manufacturers in Nigeria [5]. Further challenges identified as limiting Pharma Sector’s contribution to Nigeria’s health system, include issues with contextual capacity and suboptimal infrastructure for the production and storage of pharmaceutical products [6]. Similarly, issues relating to regulation have been fingered as a factor underpinning poor supply chain issues that impede access to medicines within the Nation’s contextual setting [7]. This is however not peculiar to Nigeria, as evidence from the World Health Organization identifies similar scenarios in a third of indicated countries across the globe [8].

Although the pharmaceutical sector represents one of the most significant industries in Nigeria [9], the challenges and capacity limitations faced by the sector, prevent it from making a commensurate contribution to National socioeconomic development. Similarly, despite representing a considerable proportion of manufacturing entities on the continent [3], these difficulties mean that only a few local pharmaceutical companies in Nigeria have the capacity to participate in international medicines’ procurement and supply. This is increasingly significant, given the commencement of the Africa Continental Free Trade Agreement [10]. Even more important, is the potential medicines’ security implication that heavy reliance on importation has, on the health and safety of a nation’s citizenry. The medicines’ security concept argues that “unless a people exert sufficient control over how their medicines and healthcare commodities are produced, sustainable access to relevant, affordable, high quality products cannot be guaranteed in that setting” [11]. Several studies have identified a potential relationship between inordinate dependence on importation, with the infiltration of substandard and falsified medicines in some developing country settings [12,13]. What this means is that Nigeria’s dependence on external courses for over half its national lifesaving medicines’ needs [4], portends significant risk for both its economy and the healthcare of its citizens.

Available evidence implies that government support is critical in overcoming a number of the factors identified as limiting the mobilisation of local manufacturing capacity to address national medicines’ requirements [11]. This approach has been identified as contextually sustainable in encouraging local pharmaceutical companies compete more favorably with their counterparts in developed countries [14]. Optimal engagement with this pathway may help address critical medicines’ security concerns relating to health access, as well as catalyse socioeconomic objectives, particularly employment generation. Other positive outcomes include capacity upgrades that will enable local companies better compete internationally thereby improving export potential, technology transfer and foreign exchange inflow [15]. If well implemented, such targeted prioritisation can create a virtuous cycle that fast-tracks the pathway to rapid industrial development in Nigeria.

In some developing countries such as India, available evidence suggests that the government regularly undertakes a proactive approach towards regulating the pharmaceutical industry whilst also providing the impetus to improve the ease of market access [16]. The implementation of policies for contextual liberalisation, establishment of public sector undertakings, and price control measures, have in the last five decades been critical in stimulating the Indian pharmaceutical industry into becoming one of the largest in the world. This is evidenced by its market size of over 40 billion USD [17,18]. This consequently contributed to achievement of medicines’ security in India. In Nigeria however, the market size of the pharmaceutical industry is valued at between 1.4–3 billion USD [19]. A review of literature revealed that within the Nigerian context, there has been little focus on gaining an empirical understanding of government’s role in driving pharmaceutical sector development. Due to significant challenges associated with a lack of medicines’ security, it is critical to develop an evidence base which can underpin contextual policies and practices aimed at improving access to essential medicines in Nigeria. This study therefore aimed at assessing stakeholders’ views and experiences on government’s role in facilitating development in Nigerian pharmaceutical sector.

## Methods

Following literature review [7,8,20–22], a data collection questionnaire (S1 File) containing questions relating to pharmaceutical sector development was developed to collect data. The questionnaire was made up of socio-demographic characteristics and items relating to pharmaceutical sector development. A Likert scale of 1 to 5 was employed for the questions which were represented as follows: 1 = Strongly Disagree, 2 = Disagree, 3 = Neutral, 4 Agree, and 5 = Strongly Agree. Face and content validations were carried out on the questionnaire using an expert panel [23]. The panel comprised 5 persons who were involved in research activities in the pharmaceutical space. The questionnaire was assessed for appropriateness, complexity, and attractiveness. Content validity ratio and content validity index tests were undertaken for each item, and only those that passed these tests were included in the final questionnaire. Cronbach alpha test was undertaken, and this gave a score of 0.763, indicating an internal consistency within the items. A pilot testing of the tool was undertaken by administering it to 10 participants that were randomly selected from practitioners in the pharmaceutical sector and the feedback received did not necessitate any major amendment.

The questionnaires were administered to the study participants who were healthcare practitioners that attended an event targeted at developing the Nigerian pharmaceutical sector. The participants were drawn from different professional areas relating to pharmaceutical sector development including policy-making, the public sector, and development agencies. Their broad representation in the pharmaceutical value chain and level of education deemed the cohort appropriate to provide insight into the objectives of the study. The data collection was undertaken in June 2022. Convenience sampling strategy was employed during data collection, and this was achieved by administering questionnaires to participants present at the event [24]. Inclusion criteria were: participants present at the conference; willingness to engage; and consent to participate in the study. Persons who did not meet these criteria were excluded from the study. A total of 82 questionnaires were administered.

Ethical approval was obtained from the National Institute for Pharmaceutical Research and Development Health Research Ethics Committee prior to the collection of data. Participation was voluntary, with informed consent obtained from the respondents before administering the questionnaires to them. Anonymity was strictly maintained during data collection, as the respondents were not required to include their names on the questionnaire, this was to ensure absolute confidentiality. The purpose of the questionnaire was appropriately explained to the study participants before administration.

Data collected were entered into Statistical Package for Social Sciences (SPSS) version 25 for statistical analysis [25]. Descriptive statistics was carried out [26], and chi square test was used to determine the association between responses and socio-demographic characteristics of the study participants. A *p*-value of 0.05 or less was considered statistically significant.

## Results

### Demography

Of the 82 questionnaires that were administered, a total of 76 were returned, indicating a response rate of 92.68%. A majority of the study participants were males (69.7%), with more than a third (38.2%) of the sample aged 51 years and above. Close to a quarter (21.15%) of the respondents had doctorate degrees. About 63.2% of the respondents were employed in the public sector. Further details about socio-demographic characteristics are presented in Table 1.

**Table 1:** Demography.

Variables n = 76	N (%)
**Gender** Male Female	53(69.7) 23(30.3)
**Age**	
18–30	9(11.8)
31–40	16(21.1)
41–50	19(25.0)
51 and Above	29(38.2)
Missing Data	3(3.9)
**Highest Education Level Achieved**	
Diploma (OND)	1(1.3)
First Degree (or HND)	29(38.2)
Master’s Degree	29(38.2)
Doctorate Degree	16(21.1)
Missing Data	1(1.3)
**Occupation**	
Public/Civil Servant	48(63.2)
Business Person	10(13.2)
Policymaker	2(2.6)
Development Agency	6(7.9)
Others	7(9.2)
Missing Data	3(3.9)

### Regulation of pharmaceutical sector

**Fig 1:**
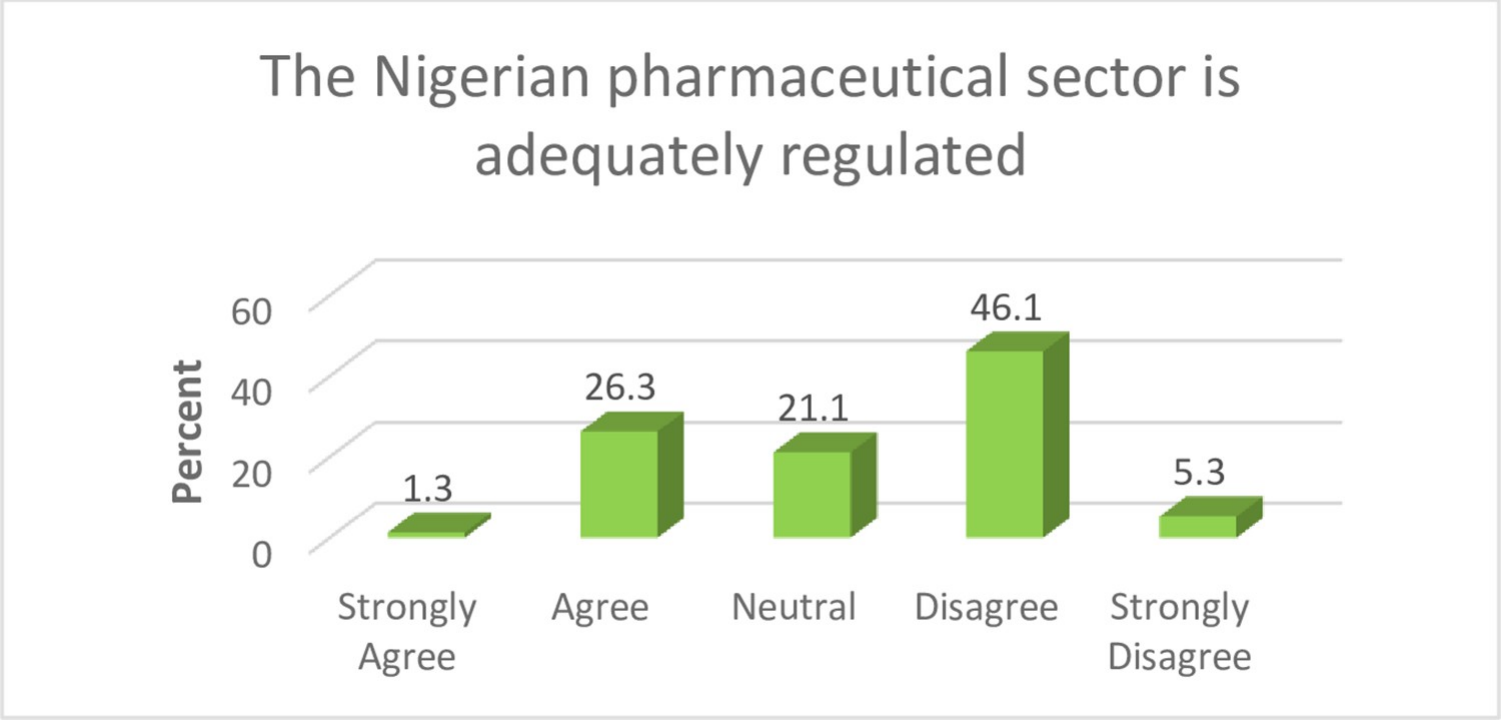
Regulation of the sector. Government retains critical responsibilities in ensuring regulation that is fit for purpose for the Nigerian contextual pharmaceutical space. Achieving an optimal level in this regard requires a robust engagement of relevant legislative, administrative, legal and technical measures aimed at ensuring the safety, efficacy, and quality of medicines.

Regulation is an important aspect of the pharmaceutical production process, in that it ensures high quality products get to consumers. It is important fo**r** the sector to be adequately regulated so as to assure the quality, safety and efficacy of medicines available within the healthcare delivery system. About half of the study participants (51.4%) indicated that there were gaps in how the Nigerian pharmaceutical sector was regulated. Further details are presented in Fig 1.

### Legislative, executive, and industry gaps

**Fig 2:**
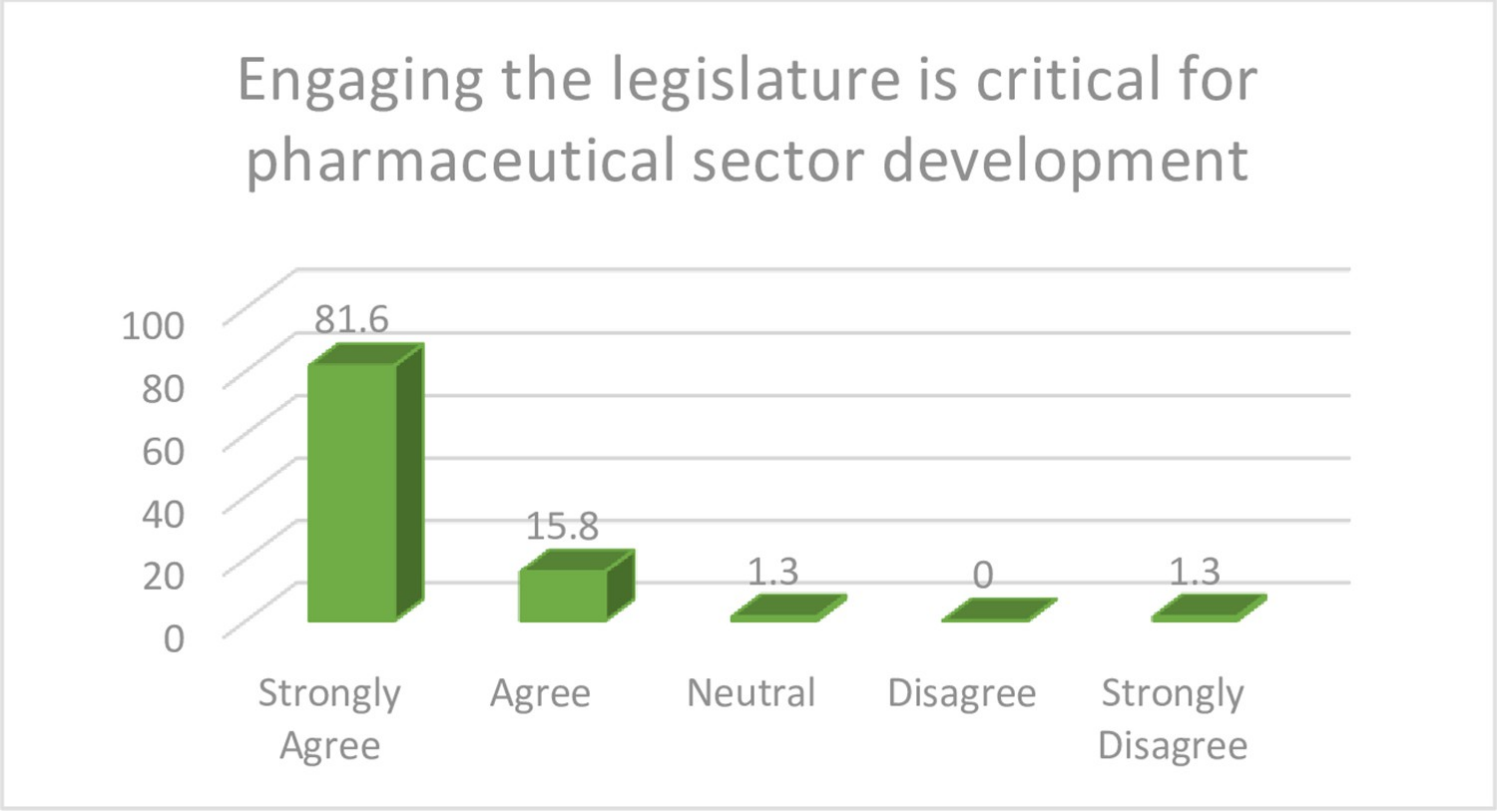
Engaging the legislature. Findings from the study suggest the need for more robust and comprehensive engagement with the legislature, especially with respect to prioritising pharmaceutical sector policies. This approach can help strengthen relevant capacities in the pharmaceutical sector in a sustainable manner.

Legislators are key stakeholders whose statutory responsibilities include representation, legislation and oversight functions. As such, their critical role in enacting and overseeing laws are considered invaluable tools that can underpin critical sectoral reforms. As presented in Fig 2 below, almost all (97.4%) the study participants indicated that the legislature should be engaged for more contextual and responsive policy making.

**Fig 3:**
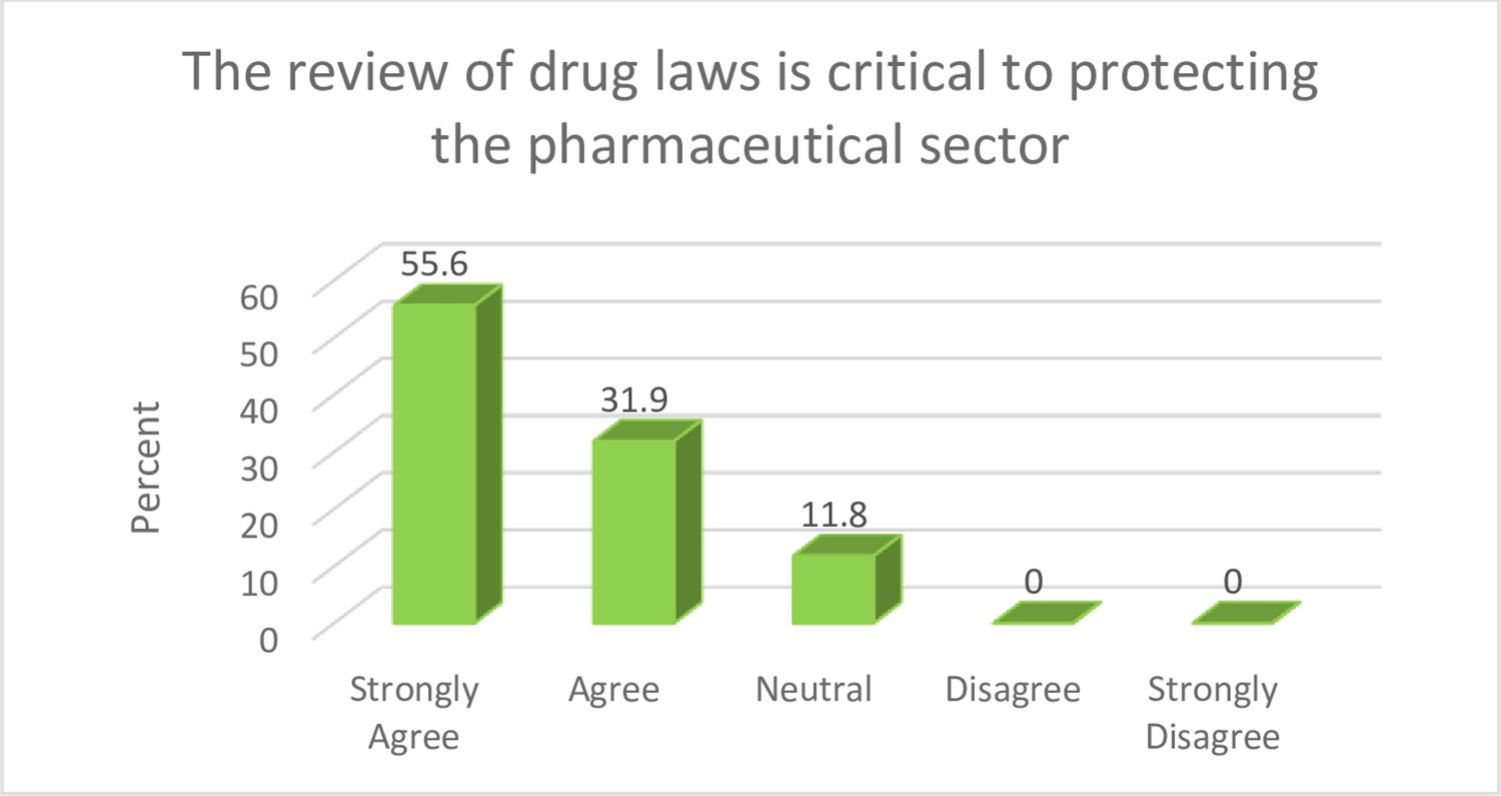
Drug law review. A major issue with existing laws and regulation is that, some specific offences and penalties to be meted on offenders are not captured. This often limits the ability of the sector regulators in undertaking their activities.

Proactive laws and policies such as regulatory harmonisation and adequate resource allocation targeted at development of pharmaceutical sector can play a prominent role in fast-tracking development in the sector. Findings from Fig 3 show that 87.5% of the study participants indicated that laws in the pharmaceutical sector should be reviewed in order to prioritise and protect the industry.

**Fig 4:**
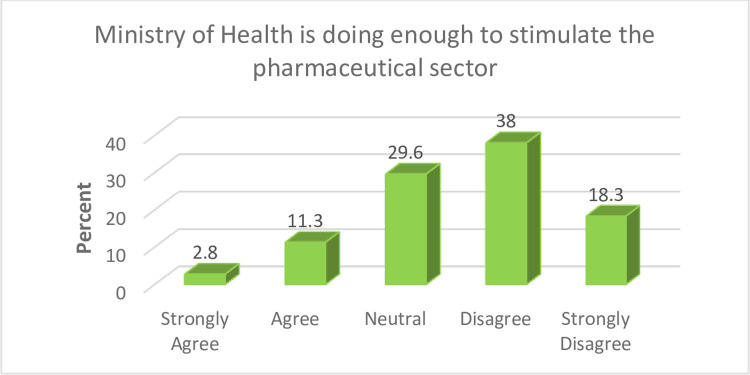
Efforts of ministry of health. This finding suggests the need for the development of contextual strategies that can expedite the growth of the Nigerian pharmaceutical sector, as this is an important strategy to achieve medicines’ security in the country.

More than half (56.3%) of the study participants indicated the need for the Ministry of Health to improve in the initiation of relevant policies aimed at stimulating the pharmaceutical sector. Further details are presented in Fig 4.

**Fig 5:**
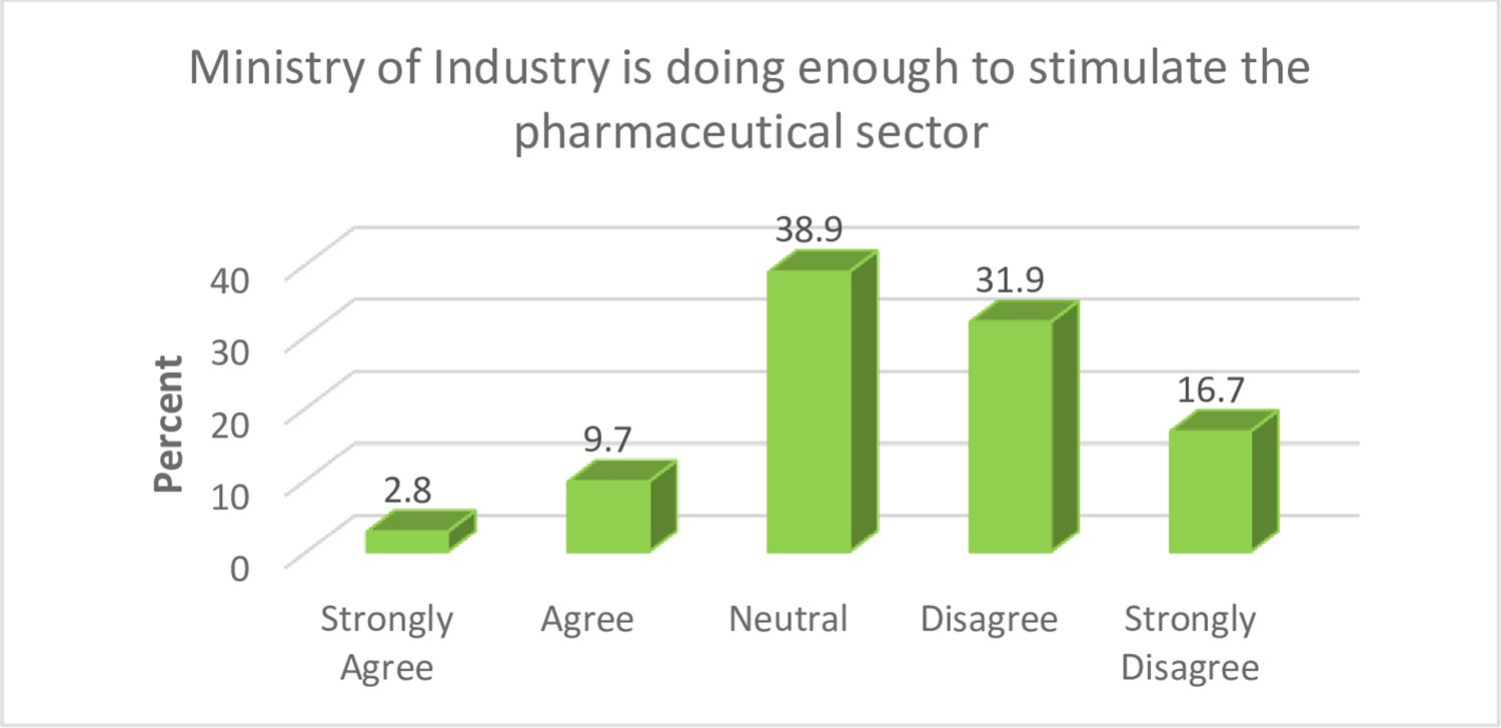
Efforts of ministry of industry. Improving the ease of doing business is an important factor that can contribute to the development of Nigerian pharmaceutical sector. The Federal Ministry of Industry, Trade and Investment needs to undertake measures such as the provision of relevant incentives and the enhancement of market access that will support investors in the pharmaceutical sector.

Finding presented in Fig 5 below shows that close to half (48.6%) of the study participants indicated that the Ministry of Industry Trade and Investment has not done enough to stimulate Nigerian pharmaceutical sector development.

**Fig 6:**
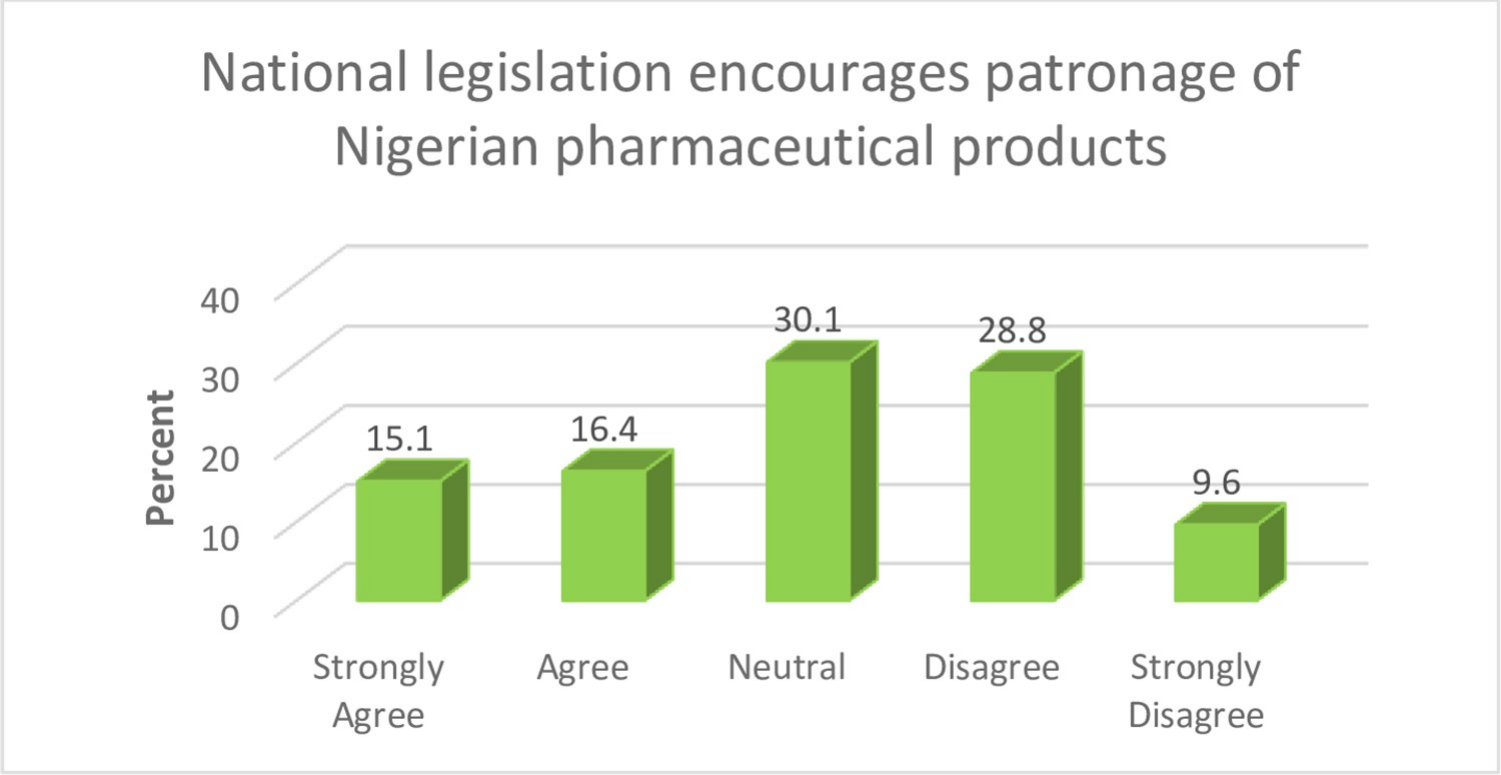
National legislation on patronage.

Patronising pharmaceutical products that are manufactured in Nigeria can serve as a way of encouraging local manufacturers in the Country. Developing contextual policies that support patronage of local pharmaceutical products in Nigeria can help ensure that policy outcomes are sustainable. However, more than a third (38.4%) of the study participants indicated that National legislation does not adequately encourage patronage. Further details are presented in Fig 6.

**Fig 7:**
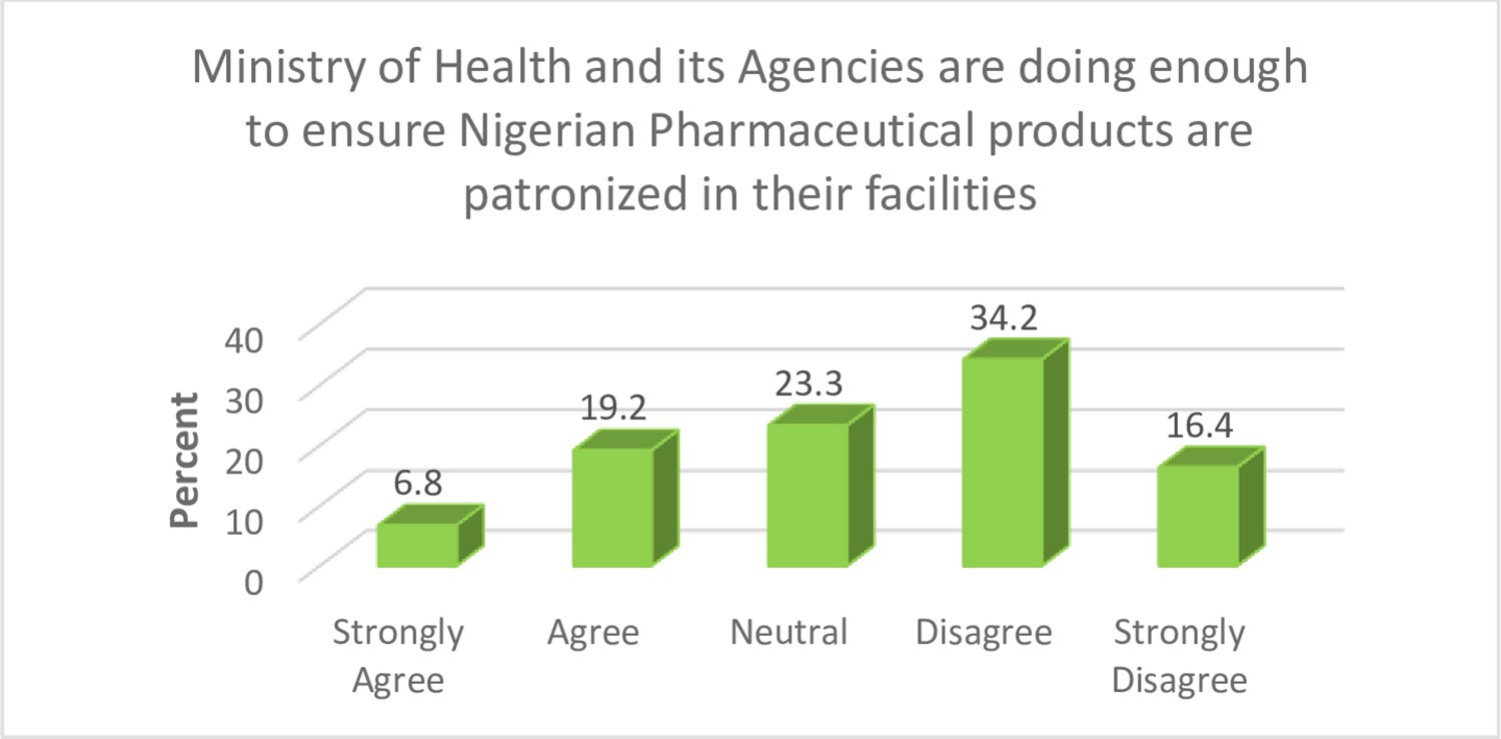
Efforts of ministry of health on patronage. This finding suggests the need for Ministry of Health to develop policy and frameworks that not only mandates healthcare facilities purchase high quality locally manufactured products, but also monitors consequent adherence.

Just over half (50.6%) of the study participants indicated that Ministry of Health and its agencies have not done enough to ensure Nigerian pharmaceutical products are patronised in their facilities. Further details are presented in Fig 7.

Further to the descriptive statistical analysis undertaken, the chi square test was also undertaken to compare relationships between variables. Findings revealed that some of the responses of the study participants were influenced by their socio-demographic characteristics. More than half of the study participants (55.6%) who were 51 years and above disagreed that national legislation encourages patronage as against 21.1% for those between 41 to 50 years, 26.6% for those between 31 to 40 years, and 44.4% for those between 18 to 30 years that disagreed (*p* = 0.015). Additionally, male participants in this study indicated support for patronage of local pharmaceutical products as 90.2% of males agreed that Nigerians should patronise local pharmaceuticals more than foreign alternatives, whilst only 65.2% of females agreed to this. Again, this finding was statistically significant (*p* = 0.033).

## Discussion

In this study, majority of the study participants indicated that the Nigerian pharmaceutical sector lacked optimum regulation. Suboptimal regulation in the sector suggests the possibility of weaknesses in access to high quality medicines in the country [27,28]. Similar findings have previously been reported in Nigeria, indicating deficiencies in the implementation and enforcement of various drug laws [29]. This is in contrast to contextual regulatory framework that exist in developed countries where implementations of relevant standards are effectively carried out [30]. The robust regulation of medicines in these countries have enabled them to achieve higher levels of access to medicines for their population compared to Nigeria and other developing nations where optimum regulation is yet to be achieved [31]. Regulation of the pharmaceutical space is a critical factor underpinning medicines’ security, since it affects the quality guarantee of pharmaceutical products available to the citizens [28,32]. There have been several reported cases of fatalities in Nigeria as a result of substandard medicines which could be attributed to lapses in regulatory activities [33–36]. Evidence from this study supports urgent regulatory reforms in the pharmaceutical sector especially, as regards monitoring and enforcement of various relevant drug laws. Capacity improvements in the areas such as manpower, logistics and technologies will strengthen overarching regulatory activities with consequent effects on access to high quality medicines. Government regulatory officials are expected to go on routine inspection of premises where medicines are manufactured so as to ensure these products comply with Good Manufacturing Practice. Previous findings however suggest a lack of adherence to statutory frequencies due to issues relating to logistics, and manpower shortages [29].

Findings from this study indicate the need for contextual legislation and policies that can prioritise and protect the pharmaceutical sector. This therefore corroborates earlier findings by Ubajaka *et al* [37] that attributed inadequate legislation as a predisposing factor to pharmaceutical products’ counterfeiting, which is a threat to medicines’ security. The indulgence in manufacturing and distribution of counterfeit medicines in Nigeria has been heightened due to the fact that penalty for the offence is lenient [35,38,39]. Ratanawijitrasin and Wondemagegnehu [40] advocated stiffer penalties for fake medicines dealers as this can make the practice less lucrative for counterfeiters. Strategies advocated in this study can build on international evidence such as the adoption of track and trace technologies and the promotion of heightened vigilance [41], to enable contextual legislation and policies that sufficiently discourage unethical practices, whilst catalysing development.

Almost all the study participants supported the need to engage the legislative arm of government in the development of pharmaceutical sector, highlighting a keen understanding of the critical role policymaking plays in catalyzing the development of the sector. This finding is in agreement with previous findings [11]. The legislature is key in policymaking, as the power to make laws is vested on this bicameral establishment [42]. This further suggests that there is a need for key players in the pharmaceutical sector to work closely with legislative arm of government for sustainable legislation and policymaking that are critical to the development of the industry. Even in developed settings, the legal and political environments directly impact all activities within the economy, as legislation is capable of determining industrial development [43]. For instance, legislation and policies on pricing and reimbursement as well as regulatory harmonization have been pivotal in developed economies to catalyze the competitiveness of the pharmaceutical industry and the effectiveness of regulation in ensuring accessibility to quality drugs [44]. These novel findings in the Nigerian setting confirm government’s acknowledged responsibilities, but also provides indicators for collaboration, initiatives and prioritization necessary for expedited pharmaceutical sector development.

Majority of the study participants indicated that the Ministries of Health and Industry had not done enough to stimulate the pharmaceutical sector, suggesting the need for urgent reformatory reviews of policies and plans for the development of the sector. Pharma related government agencies can collaborate with all key players in the sector to explore contextual strategies for development. This can improve sustainability of outcomes and also go a long way in ensuring an integrated multidisciplinary approach that can catalyse wider impact [45].

Also, in this study, more than half of the study participants indicated that government agencies have not done enough to ensure that Nigerian pharmaceutical products are patronised in their facilities. This finding provides novel evidence that may indict sector operators as regards adhering to the Executive Order Number 3, which mandates compulsory patronage for pharma products [46]. Majority of the study participants supported that Nigerians patronise more Nigerian pharmaceutical products, compared to their foreign alternatives. Widespread patronage of local pharmaceutical products can significantly increase the market share of local manufacturers, whilst simultaneously catalyzing the achievement of National socioeconomic, self-sufficiency and medicines’ security objectives. Cross-tabulation however indicated that more males supported patronage of locally manufactured pharmaceutical products, compared to their female contemporaries. Although the reason behind this is not quite clear, further insights in this area can enable more targeted campaigns that underpin better patronage of made-in-Nigeria products. Further studies therefore need to be undertaken in this area to better understand this relationship, as well as to empirically assess the level of adherence to Executive Order Number 3.

With respect to advocating legislation on patronage, interesting findings emerged, as participants were divided in their responses. Whilst a third of the study participants indicated that Nigeria legislation encourages patronage, a third of them were indefinite in their responses, and the remaining third of the study participants indicated that Nigeria legislation discourages patronage. A careful and critical assessment of extant legislation is therefore necessary, so as to better understand the extent to which these important statutes overtly or covertly influence the patronage of local pharmaceutical products.

Although this study is limited by its small cohort, the emergent findings have revealed insights regarding factors that can underpin fresh reforms in Nigerian pharmaceutical sector. The emergent evidence can begin to underpin desperately needed specific improvements, whilst further studies build on baseline findings to enable a wider framework for more sustainable interventions.

## Conclusion

This study has revealed the specific roles that the legislative and executive arms of government can play in driving development in the pharmaceutical sector. Whilst the executive is expected to provide conducive environment and necessary infrastructure needed to improve the sector, the legislative arm of government is expected to develop laws and policies that can facilitate development in the sector. The study also showed that there is a need for more collaborative working amongst all key sectoral stakeholders, in order to expedite development in the sector.

From the perspective of the study participants, it emerged that government ministries and agencies have not done enough to stimulate the development of Nigerian pharmaceutical sector. There is therefore need for government to improve on plans and strategies towards developing the pharmaceutical sector as this is critical in achieving Universal Health Coverage. Partnership efforts also need to be initiated to ensure the development of relevant capacity within the Nigerian pharmaceutical sector that will enable it not only meet National medicines’ needs, but also to compete internationally.

Contextual revision of existing laws within the pharmaceutical sector and the introduction of policies that will promote local manufacturing of pharmaceutical products needs to be given priority attention by the government. This will not only ensure the achievement of medicines’ security but would additionally catalyse socioeconomic development in critical areas such as employment generation. Emergent findings from this small study also need to be explored in further research projects, and the use of mixed methods can be considered in undertaking new studies in this area.

## Supporting information

S1 File Questionnaire. (DOCX)

S2 File Dataset. (SAV)
