# Supplementary material for: The role of government in the achievement of medicines’ security: A preliminary exploration of stakeholders’ views and experience
Source: PLoS One. 2024 Jun 7;19(6):e0299978. doi: 10.1371/journal.pone.0299978 (PMC11161072; doi:10.1371/journal.pone.0299978)
Supplement: S1 File — (DOCX) [file pone.0299978.s001.docx]

This questionnaire aimed at assessing your views in the Nigerian pharmaceutical sector to guide in Developing a Robust and Comprehensive Strategy to Catalyse development in the sector.

**Please indicate the correct option by inserting a tick in the relevant box (√), filling the dotted lines, or selecting the choice that corresponds with your level of agreement with the statements. Your responses will be anonymised and treated confidentially**

1. **Gender**

| Male |  | Female |  |
| --- | --- | --- | --- |

1. **Age group**

| 18-30 |  | 31-40 |  | 41-50 |  | 51 and above |  |
| --- | --- | --- | --- | --- | --- | --- | --- |

1. **Highest educational level achieved**

| Sec. School  Education |  | Diploma (OND) |  | First Degree (or HND) |  | Master’s Degree |  | Doctorate Degree |  |
| --- | --- | --- | --- | --- | --- | --- | --- | --- | --- |

1. **Occupation**

| Public/Civil Servant |  | Business Person |  | Politician or Policymaker |  | Development/International. Agency. |  | Other (Pls specify)  ............................. |
| --- | --- | --- | --- | --- | --- | --- | --- | --- |

**For the following statements, please indicate your level of agreement: Strongly Agree (SA), A (Agree), N(Neutral), D (Disagree), SD (Strongly Disagree).**

**5. State of the Pharmaceutical Sector**

|  | **Statement** | **SA** | **A** | **N** | **D** | **SD** |
| --- | --- | --- | --- | --- | --- | --- |
| A | The Nigerian Pharmaceutical sector is developed to international standards |  |  |  |  |  |
| B | Disharmony is an issue limiting progress in the Pharmaceutical sector |  |  |  |  |  |
| C | The Nigerian Pharmaceutical sector is adequately regulated |  |  |  |  |  |
| D | The Pharmaceutical sector has adequate manpower to drive its growth |  |  |  |  |  |
| E | Pharma sector can contribute significantly to socioeconomic development |  |  |  |  |  |

**5F Other views I have about State of the Pharmaceutical Sector are:**

**…………………………………………………………………………………………………………………………………………………**

1. **Developing a Robust & Comprehensive Strategy**

|  | **Statement** | **SA** | **A** | **N** | **D** | **SD** |
| --- | --- | --- | --- | --- | --- | --- |
| A | Engaging the Legislature is critical for pharmaceutical sector development |  |  |  |  |  |
| B | Access to medicines should be the key goal of pharma sector development |  |  |  |  |  |
| C | Government has the responsibility for driving development in the Sector |  |  |  |  |  |
| D | Increased investment in health research would stimulate the development of the pharmaceutical sector. |  |  |  |  |  |

**6E Other views and experiences I have about the Robust & Comprehensive Strategy are:**

**…………………………………………………………………………………………………………………………………………………**

1. **Developing a Pharma Sector Policy: Bridging the Legislative, Executive, Industry gap.**

|  | **Statement** | **SA** | **A** | **N** | **D** | **SD** |
| --- | --- | --- | --- | --- | --- | --- |
| A | Mutual understanding between the government and industry is critical when drafting pharmaceutical sector policies. |  |  |  |  |  |
| B | A multidisciplinary committee with representatives of all stakeholders is necessary when developing pharmaceutical sector policies. |  |  |  |  |  |
| C | Government Policies should protect the pharmaceutical sector. |  |  |  |  |  |
| D | The review of drug laws is critical to protecting the pharmaceutical sector. |  |  |  |  |  |
| E | Ministry of Health is doing enough to stimulate the Pharma sector growth |  |  |  |  |  |
| F | Ministry of Industry is doing enough to stimulate the Pharma sector growth |  |  |  |  |  |
| G | National legislation encourages patronage of Nigerian Pharma Products |  |  |  |  |  |
| H | Ministry of Health and its Agencies are doing enough to ensure Nigerian Pharmaceutical Products are patronised in their facilities |  |  |  |  |  |
| I | Nigerians should patronise Nigerian Pharmaceutical Products more than foreign alternatives |  |  |  |  |  |
| J | I personally patronise made in Nigeria Pharma Products |  |  |  |  |  |

**8G Other views and experiences I have about bridging Pharma Sector Policy gaps are:**

**…………………………………………………………………………………………………………………………………………………**

1. **Other views and experiences I have which are relevant to the theme are:**

**…………………………………………………………………………………………………………………………………………………**

**…………………………………………………………………………………………………………………………………………………**
